# An Iterative Method for Estimating Nonlinear Elastic Constants of Tumor in Soft Tissue from Approximate Displacement Measurements

**DOI:** 10.1155/2019/2374645

**Published:** 2019-01-06

**Authors:** Maryam Mehdizadeh Dastjerdi, Ali Fallah, Saeid Rashidi

**Affiliations:** ^1^Department of Biomedical Engineering, Amirkabir University of Technology (AUT), Tehran 15875-4413, Iran; ^2^Faculty of Medical Sciences and Technologies, Science and Research Branch, Islamic Azad University, Tehran 1477893855, Iran

## Abstract

**Objectives:**

Various elastography techniques have been proffered based on linear or nonlinear constitutive models with the aim of detecting and classifying pathologies in soft tissues accurately and noninvasively. Biological soft tissues demonstrate behaviors which conform to nonlinear constitutive models, in particular the hyperelastic ones. In this paper, we represent the results of our steps towards implementing ultrasound elastography to extract hyperelastic constants of a tumor inside soft tissue.

**Methods:**

Hyperelastic parameters of the unknown tissue have been estimated by applying the iterative method founded on the relation between stress, strain, and the parameters of a hyperelastic model after (a) simulating the medium's response to a sinusoidal load and extracting the tissue displacement fields in some instants and (b) estimating the tissue displacement fields from the recorded/simulated ultrasound radio frequency signals and images using the cross correlation-based technique.

**Results:**

Our results indicate that hyperelastic parameters of an unidentified tissue could be precisely estimated even in the conditions where there is no prior knowledge of the tissue, or the displacement fields have been approximately calculated using the data recorded by a clinical ultrasound system.

**Conclusions:**

The accurate estimation of nonlinear elastic constants yields to the correct cognizance of pathologies in soft tissues.

## 1. Introduction

According to the World Health Organization report, cancer is one of the principal morbidity and mortality agents throughout the world, with approximately 8.8 million deaths (nearly 1 in 6 deaths) in 2015 and 70% increase in the number of new cases over the next two decades. The cancer statistics imply the requisite to extend medical scrutiny to improve cancer prevention, early correct diagnosis, meticulous screening, and effective treatment and reduce the invasiveness and costs of applied techniques.

Since the first introduction of ultrasound (US) imaging in clinical practice in the 1970s, ultrasonography and other US modalities, for example, Doppler imaging and state-of-the-art elastography imaging methods, which provide the information related to the tissue acoustic impedance, vascular flow, and tissue mechanical characteristics or variables such as its stiffness or strain, respectively, have been extensively utilized for medical diagnoses [1]. The US imaging is recognized a noninvasive, safe, easy-to-use, low-cost, and widely accessible imaging modality for visualizing in vivo tissues. Elastography approach has currently been regarded a promising alternative to invasive medical procedures, for example, the biopsy, to characterize tissue abnormalities.

The wide variety of strategies that are being employed to quantify and image mechanical properties of biological tissues are recognized as elastography or elasticity imaging techniques with reference to their similar premise [2]:

1. The in vivo tissue is being deformed by a specified external or internal load or motion.
2. The response of tissue is being recorded by the use of a standard clinical imaging system, such as the US or magnetic resonance imaging (MRI) system.
3. The mechanical characteristics of tissue are estimated through the assessment of tissue displacement fields.

The alterations in the microstructure of tissue as a consequence of pathophysiological phenomena would change the mechanical properties of tissue; for instance, the increase in the stromal density of cancerous tissue would cause the increment in its Young's modulus [3, 4]. The outcomes of numerous experimental research studies carried out by Krouskop et al. [5], Samani et al. [6, 7], Lyshchik et al. [8], Soza et al. [9], Hoyt et al. [10], Schiavone et al. [11], O'Hagan et al. [12], and Moran et al. [13], to mention but a few, have confirmed the relation between tissue structures and macroscopic mechanical features which are being evaluated, quantified, and/or imaged by employing the palpation, elastography, digital rectal examination, and such like methods.

The precise determination of mechanical characteristics of understudy tissue by the use of an elastography technique would undoubtedly necessitate realistically appraising or modeling the tissue manners, specifically its nonlinear response to the stimulation. Hyperelasticity theory is one of the constitutive theories that have been manipulated to model the nonlinear constitutive demeanor demonstrated by biological soft tissues. A variety of hyperelastic models, for instance the well-known Neo-Hookean, Mooney–Rivlin, Yeoh, and Polynomial models, have been recommended for this purpose [14–17]. In comparison with the studies involving the linear elasticity imaging, the number of research studies conducted to image the nonlinear features of tissues is limited [2].

With the aim of diagnosing a tumor inside the understudy tissue correctly, we have utilized an iterative method, as explicated in the next section, to accurately estimate the Mooney–Rivlin hyperelastic parameters of the tumor inside the tissue. The displacement fields inside the tumor have been analyzed to extract its hyperelastic parameters. An iterative technique has been employed since it has been assumed that no initial knowledge of the tumor was accessible except the displacement fields inside the tumor. The displacement fields inside the tumor have been extracted from the simulated/recorded radio frequency (RF) signals using the cross correlation-based method. The response of an abnormal tissue to a sinusoidal load (with low frequency to negate the inertia) has been simulated by applying the finite element (FEM) software package ABAQUS. The RF signals have been simulated by the use of the Field II US Simulation Program. In brief, in this paper, we scrutinize the diagnosis of tumor through its hyperelastic parameters in the conditions where there is no primary perception of the tumor and the displacement fields inside the tumor are estimated imprecisely.

## 2. Materials and Methods

### 2.1. Hyperelasticity Theory

Constitutive theories take the improvement of mathematical models, also known as constitutive equations, into consideration in order to provide the possibility to minutely describe the behavioral characteristics of materials. Constitutive theories in continuum mechanics deal with formulating material models that are [18, 19]

- on the basis of some mechanical universal principles
- in accordance with experimental observations

Thoughtful consideration of soft tissue's model would result in the realistic prediction of its behavior such that it could be verified by experimental observations. Nonlinear constitutive manners that have been observed from soft tissues in numerous in vivo and ex vivo experimental research studies could be modeled by the use of hyperelastic models [15, 16, 20].

The hyperelastic constitutive laws deal with modeling materials with nonlinear elastic behaviors in reaction to large strains. The nonlinearities that are the consequences of (a) the material behavior and (b) the significant change in the shape of material are both regarded in the constitutive theory of hyperelastic materials. Hyperelastic materials are generally described by specific forms of strain energy density (stored energy) functions. While characterizing the homogeneous material's absorbed energy due to its deformation, the strain energy function, *W*, is defined as a function of deformation gradient, **F** [21, 22]

(1) $$\begin{array}{c} W=W\left(F\right). \end{array}$$

It is considered that B_r_ and B represent, respectively, the reference or undeformed configuration, which refers to the situations where no load is exerted to the material and the deformed configuration, which is relevant to the situations where the material is under load and therefore it may alter with time, *t*. In addition, it is assumed that **X** and **x**, respectively, correspond to the position vectors of a material point in the reference and deformed configurations, B_r_ and B. The time-dependent deformation of material, that is, the motion of material point, from B_r_ to B could be described by the function **χ**, which, for each *t*, (a) is an invertible function and (b) satisfies proper regularity conditions as follows [23]:

(2) $$\begin{array}{c} x=\chi \left(X,t\right). \end{array}$$

The deformation gradient tensor, **F**, is defined as

(3) $$\begin{array}{c} F=\mathrm{Grad}x, \end{array}$$

with Cartesian components

(4) $$\begin{array}{c} F_{i\alpha }=\frac{\partial x_{i}}{\partial X_{\alpha }}\;i,\alpha \in \left\{1,2,3\right\}, \end{array}$$

where Grad, *x*_*i*_, and *X*_*α*_ refer to the gradient operator in the configuration B_r_ and components of **x** and **X**, respectively, while the general convention,

(5) $$\begin{array}{c} J\equiv \det\;F>0, \end{array}$$

is satisfied. Due to the local invertibility of deformation, **F** should be nonsingular. The unique polar decomposition of **F** is defined as

(6) $$\begin{array}{c} F=RU=VR, \end{array}$$

where the tensor **R** is an appropriate orthogonal tensor and the tensors **U** and **V** are symmetric positive-definite tensors known as the right and left stretch tensors, respectively. Equation (7) represents the spectral decompositions of tensors **U** and **V**:

(7) $$\begin{array}{c} \begin{array}{cc} U=\overset{3}{\underset{i=1}{\sum }}\lambda_{i}u^{\left(i\right)}\;⊗\;u^{\left(i\right)}, & \lambda_{i}>0,i\in \left\{1,2,3\right\},\\ V=\overset{3}{\underset{i=1}{\sum }}\lambda_{i}v^{\left(i\right)}\;⊗\;v^{\left(i\right)}, & \lambda_{i}>0,i\in \left\{1,2,3\right\}, \end{array} \end{array}$$

where each *λ*_*i*_ refers to one of the principal stretches, **u** ^(*i*)^ and **v** ^(*i*)^ are the unit eigenvectors of **U** and **V** known as the Lagrangian and Eulerian principal axes, and ⊗ is the sign of tensor product [23, 24].

The tensor function **H** is regarded as the function of material response in the configuration B_r_ with respect to the nominal stress, **S**, that is, the transpose of the first Piola-Kirchhoff stress. The following equation for the nominal stress, **S**,

(8) $$\begin{array}{c} S=H\left(F\right)=\frac{\partial W}{\partial F}, \end{array}$$

is validated for an unconstrained homogeneous hyperelastic material. While the material is incompressible, the arbitrary hydrostatic pressure, *p*, which is the Lagrange multiplier associated with the material incompressibility, modifies the relation for the nominal stress, **S**, as

(9) $$\begin{array}{c} S=\frac{\partial W}{\partial F}-pF^{-1},\;\text{det }F=1. \end{array}$$

With regard to the relation between the nominal stress tensor, **S**, and Cauchy stress tensor, **σ**,

(10) $$\begin{array}{c} S=JF^{-1}\sigma , \end{array}$$

the Cauchy stress tensor, **σ**, could be calculated through (11) in which the symmetric tensor function **G** denotes the function of material response in the configuration B_r_ associated with the Cauchy stress tensor, **σ**,

(11) $$\begin{array}{c} \sigma =G\left(F\right)=J^{-1}F\frac{\partial W}{\partial F}. \end{array}$$

With respect to the arbitrary hydrostatic pressure, *p*, defined previously, for an incompressible material, the relation for the Cauchy stress tensor, **σ**, modifies as [23–25]

(12) $$\begin{array}{c} \sigma =F\frac{\partial W}{\partial F}-pI,\;\text{det }F=1. \end{array}$$

First, second, and third invariants of **F**, known as the strain invariants of deformation, which make provision for mapping the area and volume between the deformed configuration, B, and reference configuration, B_r_, are computed through

(13) $$\begin{array}{c} I_{1}=\mathrm{tr}\left(F\right)=F_{11}+F_{22}+F_{33},\\ I_{2}=\frac{1}{2}\left(F_{ij}F_{ij}-F_{ii}F_{jj}\right),\\ I_{3}=\det\left(F\right)=J, \end{array}$$

for an unconstrained isotropic elastic material. The left Cauchy-Green deformation tensor, **B**, and its principal invariants are calculated as follows:

(14) $$\begin{array}{c} B=FF^{T},\\ I_{1}^{B}=\mathrm{tr}\left(B\right),\\ I_{2}^{B}=\frac{1}{2}\left[{\left(I_{1}^{B}\right)}^{2}-\text{tr}\left(B^{2}\right)\right],\\ I_{3}^{B}=\det\left(B\right)\equiv {\left(\text{det }F\right)}^{2}. \end{array}$$

For incompressible materials, a slightly different set of principal invariants of **B**, as represented, is generally employed:

(15) $$\begin{array}{c} {\bar{I}}_{1}^{B}=\frac{I_{1}^{B}}{J^{2/3}},\\ {\bar{I}}_{2}^{B}=\frac{I_{2}^{B}}{J^{4/3}},\\ J_{el}=\sqrt{\det\left(B\right)}. \end{array}$$

The right Cauchy–Green deformation tensor, **C**, and its principal invariants are computed similarly.

The Cauchy stress tensor for an unconstrained isotropic elastic material is computed in terms of strain invariants, *I*_1_, *I*_2_, and *I*_3_, as follows:

(16) $$\begin{array}{c} \sigma =\alpha_{0}I+\alpha_{1}B+\alpha_{2}B^{2},\\ \alpha_{0}=2I_{3}^{1/2}\frac{\partial W}{\partial I_{3}},\\ \alpha_{1}=2I_{3}^{-1/2}\left(\frac{\partial W}{\partial I_{1}}+I_{1}\frac{\partial W}{\partial I_{2}}\right),\\ \alpha_{2}=-2I_{3}^{-1/2}\frac{\partial W}{\partial I_{2}}, \end{array}$$

as the result of the absence of *α*_0_ (because *I*_3_ = 1) and the presence of *p* for incompressible materials, the Cauchy stress tensor changes to

(17) $$\begin{array}{c} \sigma =-pI+\alpha_{1}B+\alpha_{2}B^{2}, \end{array}$$

for the forenamed materials [21–24], which simplifies to

(18) $$\begin{array}{c} \sigma =-pI+2\frac{\partial W}{\partial I_{1}}B+2\frac{\partial W}{\partial I_{2}}\left(I_{1}B-B^{2}\right). \end{array}$$

A variety of stored energy functions have been introduced in the literature that could be employed to model the nonlinear elastic behavior of soft tissues precisely [26, 27], from the popular long-standing Neo-Hookean model (originated by Treloar in 1943 [28]) and Mooney–Rivlin model (proposed by Rivlin et al. in 1951 [29, 30]) to the state-of-the-art models, for instance, the ones introduced by Limbert in 2011 [31], Nolan et al. in 2014 [32], and Shearer in 2015 (for modeling ligaments and tendons) [33], although this is hitherto an active field of study in the material and biomedical sciences.

Being pertinent to model the behavior of a wide range of materials, for instance the soft tissues and polymers, the Mooney–Rivlin model is one of the conventional hyperelastic models in the literature [34–39]. Two historical attributes have made this model a distinguished one: (1) it is one of the primarily introduced hyperelastic models; (2) it could meticulously predict the nonlinear demeanor observed from some materials, specifically the isotropic rubber-like materials. The most general version of this model has been defined based on (the linear combination of) the first and second strain invariants of deformation, *I*_1_ and *I*_2_. The Mooney–Rivlin strain energy function is expressed as

(19) $$\begin{array}{c} W=C_{10}\left(I_{1}-3\right)+C_{01}\left(I_{2}-3\right)+\frac{1}{D}{\left(J-1\right)}^{2}, \end{array}$$

where *C*_10_ and *C*_01_ are the material hyperelastic constants, *D* is the constant related to the material volumetric response (i.e., the material bulk modulus), and *J* is the determinant of deformation gradient tensor, **F**. In addition, regarding the initial shear modulus of material, *μ*_0_, the relation

(20) $$\begin{array}{c} C_{10}+C_{01}=\frac{1}{2}\mu_{0}, \end{array}$$

links the two hyperelastic parameters [26, 36]. For incompressible materials, the Mooney–Rivlin strain energy function simplifies to (since *J* = 1)

(21) $$\begin{array}{c} W=C_{10}\left(I_{1}-3\right)+C_{01}\left(I_{2}-3\right). \end{array}$$

### 2.2. Soft Tissue Simulation

To estimate nonlinear elastic parameters of an unidentified tumor using the proposed technique, we have simulated a simplified 3D breast tissue geometry utilizing the FEM software ABAQUS (Dassault Systèmes Simulia Corp., Johnston, RI, USA). The breast tissue is comprised of three partial tissues, fat, fibroglandular, and tumor, located consecutively from outside to inside. We have applied the Mooney–Rivlin hyperelastic model to evaluate the deformation of simulated breast tissue induced by the external excitation.

For estimating hyperelastic parameters of the tumor, we applied a sinusoidal load with frequency of 0.1 Hz (the very low frequency to ignore the inertia effects) to the simulated tissue and registered its response to extract the displacement fields inside the tumor. It is feasible to estimate displacement quantities inside the in vivo tissue by the use of some conventional medical imaging systems such as the US imaging or MRI system. We discuss the methods of calculating the displacements inside the tissue from RF signals or images recorded by the US imaging system in Section 2.4. In order to provide the accessibility to displacement values at some sequential moments, the US images or RF signals should be continuously saved for a period of time.

### 2.3. Simulation of US RF Signals and Images

When a tissue is inspected with the US imaging system, the tissue is scanned with respect to the probe; in other words, when the tissue is compressed with the probe, the presented features of the tissue in the image seemingly move upward, although in point of fact they would move downward. In furtherance of simulating the postcompression RF signals and B-mode images in the probe coordinate system, instead of the phantom's surface in contact with the probe, the surface in front of the probe was assigned to be moving [40–42].

The RF signals and B-mode images of the simulated phantom while responding to the sinusoidal load have been simulated using the Field II US Simulation Program (A MATLAB® toolbox for US field simulation) [43]. The nodal displacement measurements of the phantom in response to the sinusoidal load have been applied to simulate the postdeformation RF signals and B-mode images in varied deformation states. With the view to simulating the RF signals and B-mode image correlated with a particular deformation state, the correspondent nodal displacement values achieved by the finite deformation analysis have been linearly interpolated to compute the positions of scatterers corresponding to the specified deformation state.

### 2.4. Estimation of Displacement Field

In addition to the US elastography imaging techniques, motion tracking algorithms have been applied in various US-based methods, for example, blood flow imaging, thermal strain imaging, phase-aberration correction, strain compounding, and temperature imaging, to name a few. The prominence of clinical applications of motion tracking methods has contributed to the significant accrual in the number of relevant investigations and the proposal of a multitude of techniques including phase-domain tactics, time-domain (1D) or space-domain (2D) procedures, and spline-based methods [44].

Among the propounded techniques, the cross-correlation algorithm is known as the gold standard of motion estimation. In this technique, the displacement quantities are computed through searching the locations of the maximums of cross-correlation values between corresponding axial-lateral grids of small windows (with high overlap) in the pre- and postdeformation frames recorded from the medium. The shifts between the pre- and postdeformation windows quantify the displacement field in the medium [44, 45].

The cross-correlation algorithm with guided search has been applied to initially estimate the displacement quantities in the tumor at the selected step times from the simulated pre- and postdeformation RF signals and B-mode images, and afterwards evaluate the errors of displacement estimates. The calculated displacement values of proximate regions (i.e., previous samples and lines) have been exploited to reduce the search area and therefore significantly decrease the computational expense of the cross-correlation algorithm. By the use of simulated RF signals, although we devoted more time to calculate the displacement values (because of their higher sampling rate) compared with the US images, we achieved more accurate displacement estimates with higher spatial resolution.

### 2.5. Estimation of Hyperelastic Parameters

Regarding the aforementioned explanation related to the finite strain theory (also known as large deformation theory), when a uniaxial stress, **σ**, is applied to the material, the deformation gradient tensor, **F**, could be computed as

(22) $$\begin{array}{c} F=\left[\begin{array}{ccc} \lambda_{1} & 0 & 0\\ 0 & \lambda_{2} & 0\\ 0 & 0 & \lambda_{3} \end{array}\right], \end{array}$$

where *λ*_1_, *λ*_2_, and *λ*_3_ are the principal stretches with respect to the set of coordinate axes (corresponding with *x*_*i*_ = *λ*_*i*_ *X*_*i*_, *i* = 1,2,3). The principal invariants, *I*_1_, *I*_2_, and *I*_3_, could be stated further in terms of principal stretches as follows:

(23) $$\begin{array}{c} I_{1}=\lambda_{1}^{2}+\lambda_{2}^{2}+\lambda_{3}^{2},\\ I_{2}=\lambda_{1}^{2}\cdot \lambda_{2}^{2}+\lambda_{2}^{2}\cdot \lambda_{3}^{2}+\lambda_{3}^{2}\cdot \lambda_{1}^{2},\\ I_{3}=\lambda_{1}^{2}\cdot \lambda_{2}^{2}\cdot \lambda_{3}^{2}. \end{array}$$

If **λ** symbolizes the stretch parallel to the stress applied to the medium in line with the first coordinate axis (that is equal to the *λ*_1_ set), two assumptions, (1) the equality of deformations in the two other coordinate axes and (2) the incompressibility of the medium (*I*_3_ = 1), result in simplifying the relation of the Cauchy stress tensor (18), to [35, 46]

(24) $$\begin{array}{c} \sigma =2\left(\lambda^{2}-\lambda^{-1}\right)\left(\frac{\partial W}{\partial I_{1}}+\frac{1}{\lambda }\frac{\partial W}{\partial I_{2}}\right). \end{array}$$

As explicated in the previous sections, the displacement field inside the in vivo tumor could be measured in a noninvasive way. With the purpose of estimating Mooney–Rivlin hyperelastic parameters of the tumor just by using the displacement quantities, we manipulate (25), that is, the stress-stretch relation of the hyperelastic model,

(25) $$\begin{array}{c} \sigma =2\left(\lambda^{2}-\lambda^{-1}\right)\left(C_{10}+\lambda^{-1}C_{01}\right). \end{array}$$

The uniaxial load applied to the medium in alignment with the first coordinate axis practically generates the axial strain in the same direction; therefore, the strain and relation between the stress and strain could be expressed as [47, 48]

(26) $$\begin{array}{c} \varepsilon =\lambda -1,\\ \sigma =2\left({\left(\varepsilon +1\right)}^{2}-{\left(\varepsilon +1\right)}^{-1}\right)\left(C_{10}+{\left(\varepsilon +1\right)}^{-1}C_{01}\right). \end{array}$$

With respect to the stress-strain relation, (26), the iterative algorithm propounded for estimating Mooney–Rivlin hyperelastic parameters of a completely unknown interior tissue (i.e., tumor) could be described in the following steps:

(1) Image the tissue and its adjacent mediums by the use of clinical US imaging system before/while applying a sinusoidal load with low frequency (to annul the inertia) to the exterior medium. In other words, record the relevant US RF signals or images.
(2) Extract the displacement fields inside the tissue at some sequential step times (i.e., eight consecutive instants) from the recorded US RF signals or images.
(3) Simulate the tissue and its neighboring mediums by making use of one of the FEM softwares corresponding to
  (a) the recorded predeformation US images
  (b) the loading specifications
  (c) the boundary conditions
It is assumed that just the tumor and its mechanical characteristics are unidentified. The mechanical parameters of almost all healthy soft tissues have been reported in the literature. The parameters have been predominantly estimated by performing meticulous in vivo or ex vivo experiments.
(4) Consider the elastic modulus, *E*, and Poisson's ratio, *υ*, of the simulated tumor, named elastic tumor, equal 1 Pa and 0.5, respectively.
(5) Compute the displacement fields inside the particular elastic tumor at the same consecutive step times by dint of the selected FEM software.
(6) Calculate the real elastic modulus of the understudy tumor, *E*_realt_, with the help of the MATLAB^®^ software (The MathWorks, Inc., Natick, Massachusetts, USA) using
  (a) the axial displacement quantities of several points of the tumor at some consecutive instants (based on the achieved outcomes, the axial displacement values of twelve points of the tumor at eight step times), **Y**_realt_
  (b) the axial displacement values of the identical points of the elastic tumor at the same moments, **D**
  (c) the relation [47–49]
    (27) $$\begin{array}{c} E_{\text{realt}}=\frac{D^{T}D}{D^{T}Y_{\text{realt}}}. \end{array}$$
(7) Specify a set of strains, **ε**, and compute the correspondent stresses, **σ**, using (28) in accordance with (in the first iteration):
  (a) The strain field in the tumor could be roughly approximated from the displacement estimates.
  (b) The achieved results imply the selection of a set of high strains in the first iteration since the strain values are being modified in some steps of the proposed iterative algorithm; therefore, the strain values could be reduced uniformly.
  (c) Slight changes in the stress values, for instance by the use of the normal distribution function, might cause the stress and strain values to conform more effectively to the Mooney–Rivlin hyperelastic model assigned to the tumor,
    (28) $$\begin{array}{c} \sigma =E_{\text{realt}}\varepsilon . \end{array}$$
(8) Compute hyperelastic parameters of the Mooney–Rivlin model, *C*_10_ and *C*_01_, using the stress and strain sets and the relation between stress and strain, as represented,
  (29) $$\begin{array}{c} \sigma =2\left({\left(\varepsilon +1\right)}^{2}-{\left(\varepsilon +1\right)}^{-1}\right)\left(C_{10}+{\left(\varepsilon +1\right)}^{-1}C_{01}\right), \end{array}$$
  with the help of MATLAB algorithms, for instance, the regression algorithms.
(9) Assign the estimated hyperelastic parameters to the simulated tumor and compute the displacement fields inside the tumor at the determinate step times by means of the FEM software.
(10) Calculate the elastic constant of the simulated tumor, *E*_estt_, as explained previously in step 6, using the axial displacement quantities (of the appointed points at the selected step times) of the simulated tumor, **Y**_estt_, and the elastic tumor, **D** (determined in step 6), by applying the relation
  (30) $$\begin{array}{c} E_{\text{estt}}=\frac{D^{T}D}{D^{T}Y_{\text{estt}}}. \end{array}$$
(11) Appraise the estimated hyperelastic parameters for the tumor through considering the following:
  (a) The error of the computed axial displacement values of the selected points at the specified moments, **Y**_estt_, by comparing them with the correspondent displacement quantities estimated from the recorded US RF signals or images, **Y**_realt_
  (b) The error of the elastic constant calculated for the tumor, *E*_estt_, by comparing it with the real elastic modulus of the understudy tumor, *E*_realt_, estimated in step 6
The errors of the hyperelastic parameters estimated for the tumor, by comparing them with the real hyperelastic parameters of the tumor, could not be considered because it has been assumed that the tumor is entirely obscure.
(12) Alter the set of strains specified in step 7 (based on the above explanation, decrease them regularly) and repeat steps 7 to 12. By reducing the strain values steadily, the error of the estimated elastic parameter for the tumor and the error of the calculated displacement quantities in the tumor are decreasing below the defined tolerance values, as illustrated,
  (31) $$\begin{array}{c} \left\|Y_{\text{estt}}^{k}-Y_{\text{realt}}\right\|\leq e_{\text{displacement}},\\ \left\|E_{\text{estt}}^{k}-E_{\text{realt}}\right\|\leq e_{\text{elastic}}, \end{array}$$
  where *e*_elastic_ and *e*_displacement_ are the tolerance values and *k* represents the number of iterations of the algorithm. By decreasing the strain values chosen with regard to the mentioned conditions, the strain and stress values successively adjust more to the Mooney–Rivlin stress-strain relationship of the tumor.

## 3. Results

### 3.1. Soft Tissue Simulation

**Figure 1:**
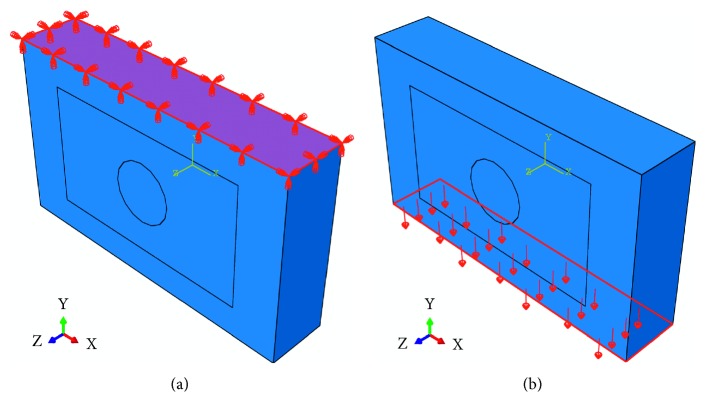
The simulated breast tissue comprises three parts, namely, fat, fibroglandular, and tumor (from outside to inside).

The breast tissue (with the dimensions of 100 × 60 × 20 mm^3^), simulated using the FEM software ABAQUS, has been depicted in Figure 1. The breast tissue consists of three partitions, namely, fat, fibroglandular, and tumor. The Mooney–Rivlin hyperelastic model has been applied to obtain the response of simulated breast tissue to the external sinusoidal load. The Mooney–Rivlin material constants of the named breast tissues have been presented in Table 1. Since we have utilized the elastic parameter of the tumor for estimating its hyperelastic parameters, we have additionally reported the elastic parameters of the named breast tissues in Table 1. The linear and nonlinear elastic parameters have been supposed to be constant throughout each tissue partition. This set of hyperelastic parameters has been broadly utilized in the literature to simulate the breast tissue [38, 50–54].

**Figure 2:**
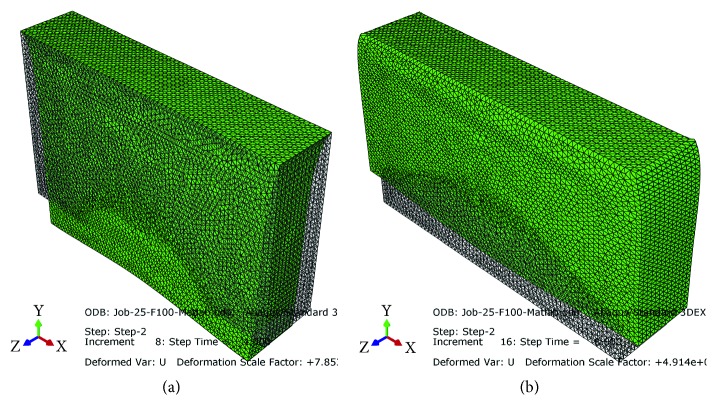
The responses of the simulated breast tissue at (a) *t* = 4.00 s and (b) *t* = 8.00 s after starting to apply the sinusoidal load.

The mesh considered for the simulated phantom consists of 183783 second-order (quadratic) tetrahedral hybrid elements (C3D10H) with 259813 nodes. The convergence analyses have warranted the accuracy of the simulation results. With reference to the explanations in Section 2.3, the number of nodes in the simulated medium should significantly be increased to precisely calculate the positions of scatterers after applying the load to the medium. With regard to the boundary conditions and the load applied to the tissue (represented in Figure 1), the postcompression RF signals and B-mode images have been simulated in the probe coordinate system. Two snapshots of the response of the simulated breast tissue to the sinusoidal load have been demonstrated in Figure 2.

### 3.2. Simulation of US RF Signals and Images

**Figure 3:**
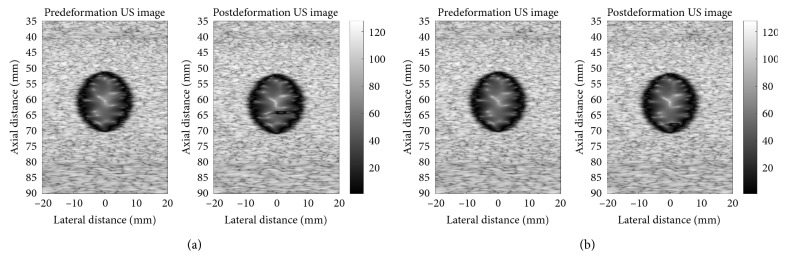
The simulated pre- and postdeformation B-mode images of breast phantom, based on its states at (a) *t* = 8.00 s and (b) *t* = 9.00 s after starting to apply the sinusoidal load.

The RF signals and B-mode images of part (with the dimensions of 50 × 60 × 10 mm^3^) of the simulated breast tissue which encircles the tumor, as illustrated in Figure 3, have been simulated using the Field II US Simulation Program. In the Field II US Simulation Program,

- The properties considered to model the probe array and simulate the US RF signals and images are as follows:
  1. Linear array (with 64 active elements)
  2. Transducer center frequency of 3.5 × 10^6^ Hz
  3. Sampling frequency of 100 × 10^6^ Hz
  4. Transmit focus of 70 mm (in depth)
  5. Element's width (the distance between the elements or the pitch of the probe array) of 0.44 mm (equal to the wavelength)
  6. Element's height of 5 mm
  7. Element's kerf of 0.05 mm
  8. Lateral spatial spacing of 0.08 mm (512 scan lines in the image)
- With regard to the elastic and hyperelastic parameters of the tumor, for scatterers which have resided within the tumor, the amplitudes are set to zero.

The postdeformation RF signals and B-mode images in eight deformation states of the phantom (corresponding to eight sequential step times: 7.75 s, 8.00 s, 8.25 s, 8.50 s, 8.75 s, 9.00 s, 9.25 s, and 9.50 s, while responding to the sinusoidal load) have been simulated using the displacements of the phantom's nodes computed by the finite deformation analysis. The nodal displacement values correlated with a particular deformation state have been linearly interpolated to compute the positions of scatterers and simulate the correspondent RF signals and B-mode images thereafter. Two simulated postdeformation B-mode images (pre- and postdeformation images) associated with the step times of 8.00 s and 9.00 s have been represented in Figure 3.

### 3.3. Estimation of Hyperelastic Parameters

After simulating the pre- and postdeformation RF signals and B-mode images correlated with the defined deformation states, the cross-correlation algorithm with guided search, as briefly described in Section 2.4, has been employed to initially estimate displacement quantities inside the tumor at the selected step times and afterwards evaluate the errors of displacement estimates.

The suggested iterative algorithm, comprehensively explicated in Section 2.5, has been applied to extract the Mooney–Rivlin hyperelastic parameters of the tumor from the axial displacement values of some points of tumor at the specified step times. As represented in Table 2, precise estimates of hyperelastic parameters of the tumor have been achieved. The automatic iteration of the algorithm would be feasible through bilaterally connecting the MATLAB and FEM softwares.

## 4. Discussion

In the majority of diversified approaches proffered for estimating elastic parameters of soft tissues, particularly the nonlinear ones, for instance, the techniques proposed by MacManus et al. [55], Esmaeili et al. [56], Omidi et al. [57], Roy and Desai [58], Liu et al. [59], Boonvisut and Çavuşoğlu [60], and Wang et al. [61], to mention but a few, the alterations (of precise values) of at least two deformation variables, which are associated with the mechanical characteristics of soft tissues, have been exploited. The assessment of recommended techniques would reveal that the direct dependencies of methodologies to deformation variables except the displacement (and strain) have impelled the researchers to carry out experiments on ex vivo tissues, or perform invasive procedures to precisely measure the variables; consequently, the emphasis of recent investigations should be on advancing noninvasive methods with the capability to accurately estimate nonlinear elastic parameters of tissues.

The hyperelastic constitutive theory takes two types of nonlinearities perceived in responses of soft tissues, into consideration [62, 63]:

- The material nonlinearity of the stress-strain relation, known as the physical nonlinearity
- The nonlinearity of the strain-displacement relation, called the geometrical nonlinearity

consequently, it has been regarded as one of the best practical theories for formulating mechanical behaviors of soft tissues. To the best of our knowledge, amongst the strategies proposed for quantifying hyperelastic parameters of materials, two methods founded on the displacement fields inside and on the boundary of the medium (i.e., phantoms) which have been introduced by Mehrabian and Samani [26, 27, 64] and Hajhashemkhani and Hematiyan [47, 48], respectively, could be utilized to noninvasively reconstruct hyperelastic parameters of in vivo tissues. The displacement field inside the understudy medium could be extracted from RF signals or images recorded by a clinical US imaging system, for instance, by using the conventional cross-correlation method.

In the technique recommended by Mehrabian and Samani [26, 27, 64], the displacement quantities of a large number of contiguous points inside the medium should be utilized to calculate the defined coefficient matrix which correlates the stress distribution computed for the tissue (with the help of a finite element model of the tissue deformation) to its hyperelastic parameters. The displacement values at some boundary points of the understudy medium have been manipulated by Hajhashemkhani and Hematiyan [47, 48] for characterizing its nonlinear material constants.

On account of the explanations provided by Mehrabian and Samani [26, 27, 64] and Hajhashemkhani and Hematiyan [47, 48] and the results achieved through the implementation of their methods (part of them published in our paper [65]), it has been realized that precise estimates of hyperelastic parameters of the understudy medium could be attained through the following:

- Accurately calculating the displacement quantities, respectively, in a multitude of adjacent points of the medium and in several boundary points, which might not be possible using registered US images or RF signals
- Applying proper regularization techniques, for instance, the Tikhonov regularization, Truncated Singular Value Decomposition (SVD), and Wiener Filtering methods
- Even considering appropriate initial guesses of the hyperelastic parameters, as indicated by Hajhashemkhani and Hematiyan [47, 48], Aghajani et al. [66], and Kim and Srinivasan [67]

The limitations of the techniques propounded with the aim of reconstructing nonlinear elastic parameters of in vivo soft tissues persuade us to concentrate on developing a more practical method with consistent results. Our primary upshot represented in Section 3 confirms that accurate estimates of hyperelastic parameters of the understudy tissue (i.e., tumor) could be obtained on the basis of the displacement values of some points inside the tissue, which has been excited by a low frequency sinusoidal load, even when there is no prior knowledge of the tissue.

The displacement quantities of the selected points might be computed approximately, for instance, by the use of the cross-correlation technique as a consequence of recording low-quality US images or RF signals or other attributes; therefore, we have evaluated the consistency of calculated values for the hyperelastic constants by applying errors with normal distribution to the measured displacement fields in the tissue stimulated by the sinusoidal load. At this point, the average errors of the displacement values estimated for the selected points from the simulated US RF signals and images using the cross-correlation algorithms (without/with guided search with respect to the calculated displacements of previous lines or samples) have been regarded. The achieved results have been demonstrated in Table 3.

It should be considered that the imprecise estimates of displacement fields inside the tumor affect all the computed parameters and errors, even the real elastic modulus of the understudy tumor; therefore, the results presented in Table 3 could not be compared. Similar to the case where the displacement values of the appointed points are exact,

- the error of calculated axial displacement values of the selected points at the specified moments, **Y**_estt_
- the error of elastic constant computed for the tumor, *E*_estt_

(as explained in step 11 of the proposed algorithm in Section 2.5) have been considered except in the situations where the displacement errors are significant.

The convergence of the aforementioned errors to values, which might not be small errors, specifies the best estimates of hyperelastic parameters when the displacement values are highly inaccurate, as represented,

(32) $$\begin{array}{c} \left\|Y_{\text{estt}}^{k}-Y_{\text{estt}}^{k-1}\right\|\leq e_{\text{displacement}}^{\prime },\\ \left\|E_{\text{estt}}^{k}-E_{\text{estt}}^{k-1}\right\|\leq e_{\text{elastic}}^{\prime }, \end{array}$$

where *e*_elastic_′, *e*_displacement_′, and *k* are, respectively, the specified tolerance values and the number of iterations of the algorithm. Provided that the set of strains (required in step 7 of the algorithm described in Section 2.5) is selected properly based on the displacement fields calculated for the tumor, precise estimates of tumor's hyperelastic parameters could be obtained. With regard to the outcomes summarized in Table 3, it is deduced that the suggested method is strongly resistant to the displacement errors.

The US RF signals recorded by means of the Antares Siemens system (Issaquah, WA) at the center frequency of 6.67 MHz from an elastography phantom (CIRS elastography phantom, Norfolk, VA) have been utilized to evaluate the suggested method experimentally. The signals were registered via a VF10-5 linear array at a sampling rate of 40 MHz by Rivaz et al. to assess the performance of the proposed real-time static elastography techniques which were based on the analytic minimization of regularized cost functions. Young's moduli of the lesion and surrounding medium have been reported 56 kPa and 33 kPa, respectively, while the phantom is under compression [68, 69].

The enhanced cross-correlation algorithm, in that the search regions were minimized with respect to the estimated displacements of previous lines or samples, has been employed to compute the axial displacement field in the compressed phantom. The Kalman filtering, introduced by Rivaz et al. [68], has been applied to calculate the strain field in the compressed phantom from the displacement measurements. Minor differences between the displacement fields estimated by the use of the enhanced cross-correlation algorithm and analytic minimization method validate the results of the former technique.

**Figure 4:**
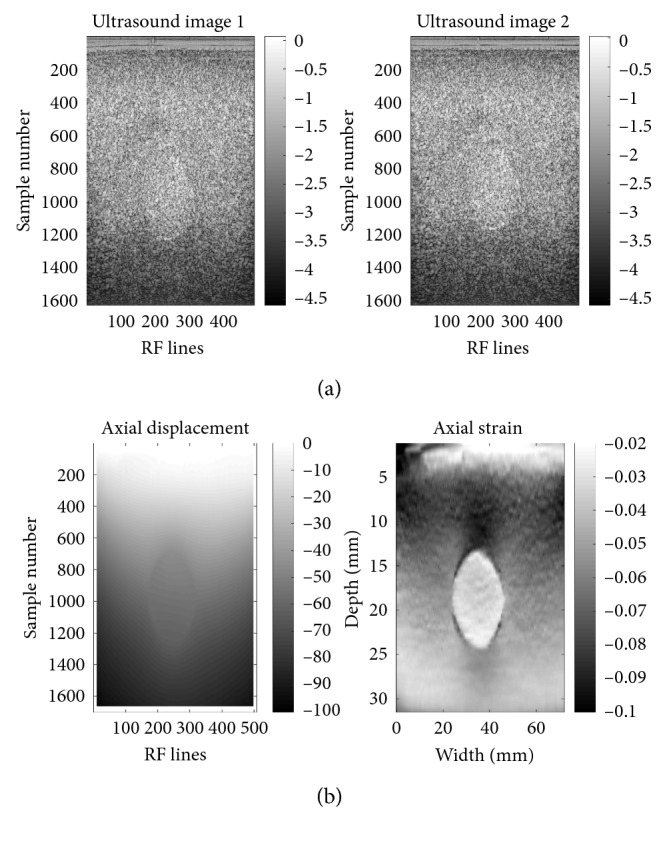
(a) Two US images of the phantom and (b) the estimated axial displacement and strain fields. The recorded RF signals of the elastography phantom (by Rivaz et al. [68, 69]), before/after it was compressed, have been used to create the US images and compute the axial displacement and strain fields.

The US images of the phantom constructed from the recorded RF signals and the estimated displacement and strain fields have been represented in Figure 4. Following the instructions in Section 2.5, the elastic and hyperelastic parameters of the lesion could be calculated from the estimated axial displacement and strain fields in the lesion. The percent error of the elastic parameter computed for the lesion, on the basis of the explanations in step 6 of the algorithm, is 15.06%; in other words, *E*_realt_ has been estimated 47565.72 Pa. The relation between stress, strain, and the parameters of the Mooney–Rivlin hyperelastic model, *C*_10_ and *C*_01_, has been manipulated to compute the hyperelastic parameters of the lesion. The values of 6871.65 Pa and 1020.00 Pa have been obtained for the mentioned parameters.

On the basis of the achieved results summarized in Sections 3 and 4, it is concluded that the main objectives that have been accomplished in this paper are as follows:

- The identification of an entirely unknown tissue (i.e., tumor) in a soft tissue through its nonlinear elastic parameters
- The feasibility to use imprecise measurements of displacement in the tumor, which are extracted from the signals or images recorded from the soft tissue while responding to a sinusoidal stimulus (with low frequency)

## 5. Conclusion

In this paper, the noninvasive diagnosis of tumors in soft tissues, such as the breast, through their nonlinear elastic parameters has been evaluated by the use of a novel iterative algorithm founded on the principle of US elastography technique. The achieved results could be undoubtedly considered the validation of the precise estimation of hyperelastic constants of an undiagnosed pathology which has been accomplished:

- By manipulating the relation between stress, strain, and the parameters of a hyperelastic model as explicated in the paper
- Based on the response of tissue to the sinusoidal load with low frequency, indeed the displacement quantities of a few points of tissue at certain step times

The displacement fields inside the tissue could be noninvasively computed from the data recorded by the employment of conventional medical imaging modalities, for instance, the RF signals or images registered by the US imaging system. Even by processing approximate displacement measurements, accurate estimates of the material constants could be obtained. The competency of the proposed method to estimate nonlinear elastic constants of normal and abnormal in vivo tissues will be further appraised in the future research.

## Figures and Tables

**Table 1:** The elastic and Mooney–Rivlin hyperelastic constants of breast tissues [38].

Hyperelastic and elastic parameters	Fat	Fibroglandular	Tumor
*C*_10_ (Pa)	2000	3500	10000
*C*_01_ (Pa)	1333	2333.3	6667
*E* (kPa)	20	35	100

**Table 2:** The elastic and hyperelastic parameters estimated for the tumor.

Estimated elastic and hyperelastic parameters		
2 estimates		
*E*_realt_ (kPa)	88908.41	
Error of *E*_realt_ (%)	11.09	
*C*_10_ (Pa)	9426.98	10005.05
*C*_01_ (Pa)	6702.30	5992.82
Error of *C*_10_ (%)	5.73	0.05
Error of *C*_01_ (%)	0.53	10.11
*E*_estt_ (kPa)	88711.93	88658.27
Error of *E*_estt_ (%)	0.22	0.28
Error of **Y**_estt_ (%)	0.28	0.35

**Table 3:** The hyperelastic parameters estimated for the tumor using inaccurate displacement measurements.

Estimated hyperelastic parameters	Inaccurate displacement measurements			
	Error 2%	Error 5%	Error 8%	Error 10%
*C*_10_ (Pa)	9386.78	9484.46	9815.78	9858.99
*C*_01_ (Pa)	6889.50	6817.80	6437.30	6465.64
Error of *C*_10_ (%)	6.13	5.16	1.84	1.41
Error of *C*_01_ (%)	3.34	2.26	3.45	3.02
Error of *E*_estt_ (%)	0.53	0.39	1.89	2.29
Error of **Y**_estt_ (%)	1.77	4.59	6.28	7.96

## Data Availability

Since the research is still in progress, the authors have decided to make data available upon request.
